# Neural population coding of movement direction for path integration

**DOI:** 10.1186/1471-2202-14-S1-P211

**Published:** 2013-07-08

**Authors:** Jaehong Lee, DaeEun Kim

**Affiliations:** 1 Biological Cybernetics, School of Electrical and Electronic Engineering, Yonsei University, Shinchon, Seoul, 120-749, South Korea

## 

Many insects including desert ants (*Cataglyphis fortis*) and honeybees can return their home after foraging. Desert ants choose a direct path to return home after a long journey, and it is believed that they use path integration approach. Path integration process needs internal integrator to accumulate their traveled distance and direction to estimate the homing vector. Biologists showed that *Cataglyphis fortis *uses a step integrator as an odometer [1]. The path integration system of the desert ants has an interesting aspect, and Mueller and Wehner [2] showed that there are errors of path integration in two-leg trajectory experiments and their polar coordinate system model fits well to the experimental data. Homing direction error may be systematic and with these estimation errors, desert ants may find their nest efficiently by combining egocentric information and visual information including landmarks in the environment. By observing the desert ant's estimation of distance travelled, Sommer and Wehner [3] found that desert ants underestimate their travelled distance. These underestimated errors grow as their travel distance increases. They tried to fit these underestimated distances versus travelled distance results with some model functions: linear, power, logarithmic and exponential. Among these model functions, logarithmic and exponential models show good fit with experimental data (Sommer and Wehner, 2004). Also Merkle et al. [4] proposed an egocentric polar coordinate model to explain the leaky integrator of the desert arthropods. Wittmann and Schwegler [5] showed a neural network model for path integration, which has a population coding of neurons for movement direction. Here we introduce a new neuronal model for path integration system. It includes the first-order low-pass filter for a leaky integrator and a population coding of neurons with a circular array of direction neurons. The movement direction is encoded in the population coding, which uses allocentric information from a polarized light. An exponential decay term for travelled distance is now expressed with a low-pass filter, which can be easily implemented with a simple neuron. A circular array structure of neurons, where each unit represents a movement direction, can encode the movement direction as a form of population coding, and the integration over neuron activations includes the accumulated path for each direction. A decoding process over those neuron activations can estimate the homing direction as well as the homing distance. Each neuron unit encompasses a leaky integrator system as often observed in a neuronal process. Our experiments show that this leaky integrator system triggers errors in two-leg trajectory movements of an agent, and our simple model fits well to the experimental data for two-leg trajectory experiments. This systematic error in path integration can help desert ants easily find their home with visual landmark navigation. We assume a circular array of directionally sensitive neurons to explain the path integration system of desert ants, but what type of neural structure is available in a real neuron system of desert ants or what kind of neural interactions in a circular array of neurons is needed for the path integration is an open question. We need further study on these subjects. More sophisticated models and experiments might explain those questions.

**Figure 1:**
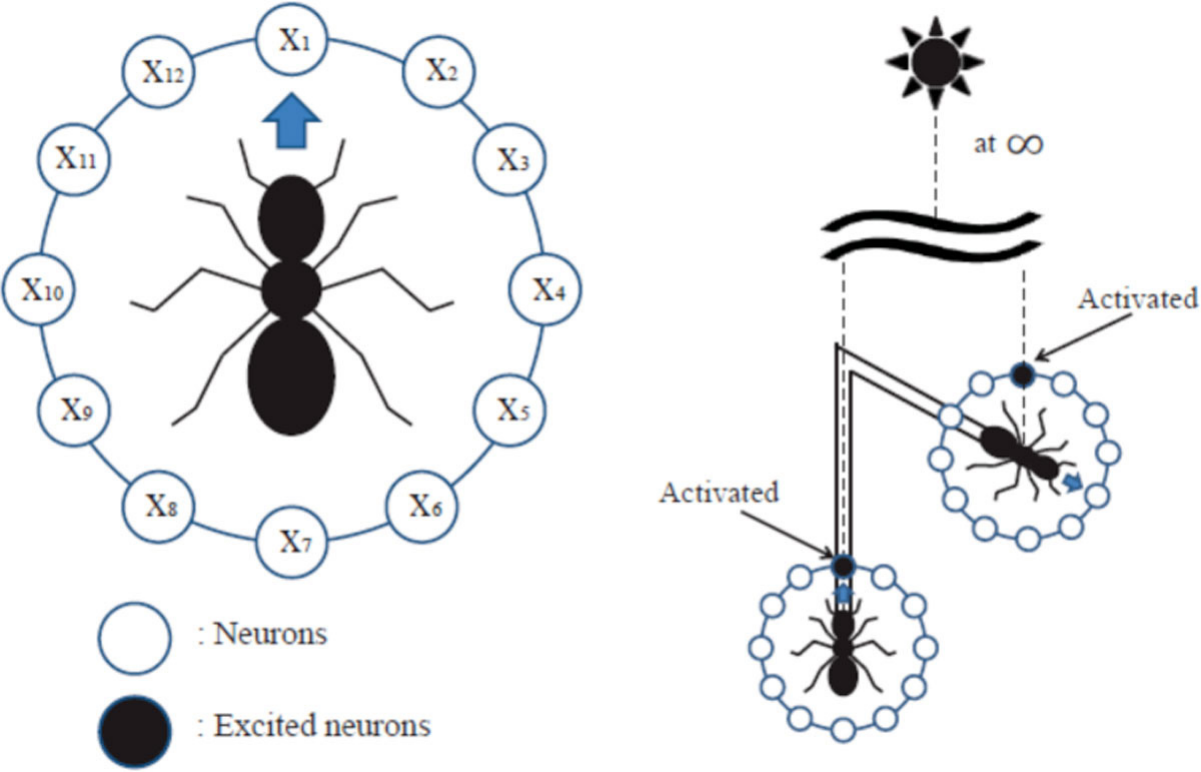
**Diagram for neuronal population coding of movement direction**.
